# The Actual Cautery

**Published:** 1881-10

**Authors:** T. F. Frank

**Affiliations:** Pittsburgh, Pa.


### ARTICLE II.

THE ACTUAL CAUTERY. By T. F. FRANK, M.D., Pittsburgh, Pa.

Within the meaning of the term actual cautery are embraced all methods by which animal tissues are destroyed, by contact with heated instruments, whether the heat is produced by fire directly, or by the aid of the galvanic battery; and the importance of the application consists in the quality of temperature, but not the mode by which it is produced. By the selection of galvanism for the heating of cautery instruments, however, we are better able to secure to them a more uniform temperature, essential for a successful issue of those operations, than can be done by any other process, and so far the galvanic battery has advantages. Its electric influence is no other than that obtained from metal heated by fire.

With all the advantages the electro cautery can be claimed to possess, it is highly overestimated, and not worth the money for an outfit.

During the past twelve years, I have had ample opportunities to test all the so-called modern improvements in galvanic cautery apparatus in operations on cancers, polypi, hæmorrhoids, and in uterine surgery, but I must confess that many times, when my expensive fancy instruments failed me, I had to fall back upon a clumsy carbon-zinc cell made for me years ago, by an ordinary mechanic, at a moderate price.

Two years ago Mr. Isaac G. H., of Bradford, McKean Co., Pa., presented himself at my house for an operation on piles by galvano-cautery. The tumors were unusually large, and I feared that the Dawson battery I owned would fail me, as it had done in minor cases; and being unwilling to transport the large cell before mentioned to the hospital where the patient had taken a room, I obtained, through the kindness of Dr. D. N. Rankin, of Allegheny City, a Byrne battery, to help me out should the Dawson fail. Both the instruments were in good condition, and produced all the heat any manipulator could obtain from them; but had it not been for the most economical expenditure of the battery force, I could not have completed the operation. Much of the successful issue of this case depended upon the service I obtained through the assistance of Drs. Rankin and R. S. Sutton, which enabled me to give attention to the action of the batteries, which otherwise I would have been obliged to trust to more inexperienced hands.

Some time in July, 1880, I was requested by Dr. Willard, of Allegheny City, to remove for a patient of his, from Toledo, Ohio, the greater part of the neck of the uterus, on account of carcinoma involving the os. The doctor had just purchased a Piffard cautery, and wishing to test its capacity, requested me to use it in this case. I fortunately made some experiments with this battery, after having prepared it according to directions accompanying the instrument, or the operation would have been a failure. The knife to be employed became hardly warm, while I used the manufacturer's formula for acid, but subsequently I obtained better results, and had the patient prepared for the operation. With a far stronger solution than directed by the maker of the battery, I could heat the platinum knives very well, but was unable to maintain a good temperature during the operation, and there was considerable hæmorrhage to contend with.

I consider it bad practice to rely upon the actual cautery as an instrument for the removal of any growth to which access can be had by the knife, the scissors, or any other rapid method of extirpation. The surgeon does not contemplate dealing with the parts to be removed, but to bring the remaining tissues in the most favorable condition for recovery, and for that purpose the actual cautery becomes a most valuable remedy.

There are no tumors to which the actual cautery is applicable but what any ordinary surgeon may remove with the knife first, and control the bleeding temporarily, till the cautery can be applied. In cases of piles, cauterization is most eminently useful, and in all those cases the bleeding may be prevented by clamping the tumor before cutting it off, and before the cautery can be brought to action.

Now comes the consideration, whether for the cauterization of an open, accessible surface, we need the complicated and expensive paraphernalia connected with our electro-cautery instruments, or whether that operation cannot be more simplified? Every surgeon, no matter how obscure his practice, will find occasions to use the actual cautery, but few are able to purchase a battery, at an expense of $60—or $85 with the necessary cautery instruments—and at that obtain a very unreliable apparatus, probably never to be used after the first attempt.

Acting upon this suggestion, I have constructed a more simple cautery, which is in every particular reliable, and can be furnished for one fifth the cost, including the burner, for what any electro-cautery battery is sold without the handles, domes and knives.

For the benefit of those who wish to interest themselves in that particular practice, I give the following description of my new cautery, which I have named the Hydrogen Cautery: It consists of a simple closed copper and zinc element, so arranged internally that the hydrogen escaping from the negative element is retained inside the cell, and is rapidly acquiring density, till a pressure of three ounces to the square inch is obtained. By that time the zinc is free from the contact with the acid, and no more gas can be generated till a portion is allowed to escape for the purpose of cauterization.

The instrument has the capacity of one quart, and after being charged with eight ounces of water acidulated with four ounces of common sulphuric acid, it will perform steady cautery work upon the most bloody tissues during one hour. The *modus operandi* of this cell, after the acid has been introduced, is perfectly automatic.

For the cautery I have constructed a small Bunson burner out of pipe clay, which with the full pressure of the gas will produce an almost invisible flame the size of a pepper-corn. The caliber of it can be reduced to that of a pin's head. The latter I adopt in most all of my operations.

Mr. William S. S., of Duke Center, Pa., was sent to me for an operation on piles, by Dr. Thos. Stuart, of Erie, Pa. He came at the time when I was making comparative experiments in the presence of several medical gentlemen with the Piffard and Dawson batteries and my hydrogen cautery upon raw beef. The experiment conclusively demonstrated that with one charge of my cell, I could cauterize more surface than the other two did unitedly. The two batteries had to be shaken and pumped constantly, and the acid liquid became boiling hot, while I had my cell in the side pocket of my coat, perfectly cool, and performing its work admirably. Mr. S. was operated upon the next day. He had five very large piles. After constricting the tumors by a snare clamp I lately constructed for those operations, I cut them down with a bistoury, and then simply brushed the little flame, held at a proper distance, over it, till the stumps were as dry and of the appearance of sponge. I never obtained as good results from a cautery operation before this. The patient recovered without requiring any attention after the operation, and left the city on a visit to his relatives in Michigan the sixth day.

For the more delicate operations on the cavities of the mouth and nose, where it becomes of great importance to confine the heat to a particular spot during a definite period, to prevent organs like the Eustachian tube and the middle ear from suffering from the heat radiated by the cautery, I have arranged an electric device, by which I can instantly send a small jet of heat, after the burner has been applied cold to the diseased part, under cover of a shell. This maneuver can be accomplished by the motion of one finger, and the heat stopped instantly in a like manner.

I give this paper with a desire to disincumber the actual cautery, and popularize one of the most promising surgical appliances.

